# Carbon reduction technology pathways for existing buildings in eight cities

**DOI:** 10.1038/s41467-023-37131-6

**Published:** 2023-04-04

**Authors:** Yu Qian Ang, Zachary Michael Berzolla, Samuel Letellier-Duchesne, Christoph F. Reinhart

**Affiliations:** Massachusetts Institute of Technology, Cambridge, MA 02139 USA

**Keywords:** Energy efficiency, Energy modelling, Energy management, Energy policy, Energy conservation

## Abstract

We work with policymakers in eight cities worldwide to identify technology pathways toward their near- and long-term carbon emissions reduction targets for existing buildings. Based on policymakers’ interests, we define city-specific shallow and deep retrofitting packages along with onsite photovoltaic generation potential. Without further grid decarbonization measures, stock-wide implementation of these retrofits in the investigated neighborhoods reduces energy use and carbon emissions by up to 66% and 84%, respectively, helping Braga, Dublin, Florianopolis, Middlebury, and Singapore to meet their 2030 goals. With projected grid decarbonization, Florianopolis and Singapore will reach their 2050 goals. The remaining emissions stem from municipalities not planning to electrify heating and/or domestic hot water use. Different climates and construction practices lead to varying retrofit packages, suggesting that comparable technology pathway analyses should be conducted for municipalities worldwide. Twenty months after the project ended, seven cities have implemented policy measures or expanded the analysis across their building stock.

## Abstract (web summary)

Here, the authors perform analysis of technology pathways for existing buildings using urban building energy models developed with cities, showing that shallow and deep retrofits along with onsite photovoltaic and grid decarbonization can help achieve carbon reduction targets.

## Introduction

Cities run on energy. Since the industrial revolution, urban environments have dominated energy consumption patterns in countries around the world. Today, over 50% of the world’s population lives in urban areas, collectively generating over 75% of the global gross domestic product (GDP). Attracted by this wealth, urban dwellers are expected to double by 2050. At that point, the urban built-up area is projected to more than triple^1^, accounting for over 70% of global carbon emissions^2^.

Cities are well positioned to mitigate future emissions, being both “a cause of and solution to” climate change^3^. More than 100 cities have already committed to net-zero carbon emissions by 2050^4^. Buildings will inevitably play a pivotal role in this process, with the Intergovernmental Panel on Climate Change (IPCC) estimating that the energy use of existing residential buildings can be reduced by 50% to 75% in many geographical regions^5^. However, while the long-term goals are clear, the pathways to achieving carbon emissions reduction targets for buildings are less so. To keep cumulative carbon emissions of the global building stock in check, the annual global renovation rate must increase from (the current) 1% to 5%, and all new construction must be carbon neutral by 2040 in terms of both operational and embodied energy use^6,7^. For The United Kingdom, this translates into a retrofit rate of 1.5 homes every minute from now until 2050^8^. In the United States, the Biden administration has supported energy efficiency upgrades in at least four million homes and weatherization for at least two million homes^9^. To optimize the use of such funds, cities need a data-driven support framework to decide what type of upgrades to encourage and in what buildings.

In this paper, we focus on technology pathways to reduce annual carbon emissions in existing buildings based on retrofitting measures and onsite rooftop photovoltaics (PV). Our analysis includes long-term local grid decarbonization targets but does not consider socioeconomic factors such as willingness-to-pay models^10^, stock turnover, or new construction. We focus on retrofitting opportunities in existing buildings as a foundational first step for city governments to consider before pursuing other carbon emission reduction strategies, including carbon offsets, etc. Parallel analysis is necessary to evaluate emissions impacts from industry, transport, land use, and new construction. For the latter case, unless all new buildings are built to net-zero standards starting today, the challenge cities face in meeting their building-related emissions reduction goals will only be heightened.

We collaborated with representatives from eight cities and municipalities around the world—Braga (Portugal), Cairo (Egypt), Dublin (Ireland), Florianopolis (Brazil), Kiel (Germany), Middlebury (Vermont, United States), Montreal (Canada), and Singapore. The cities were selected based on public calls for participation on building/urban science mailing lists and via direct contacts in our networks. A requirement for participation was that teams had some expertise in building energy modeling as well as existing relationships with local city representatives. We also aimed for a diverse set of cities with different climates, socioeconomic demographics, cultures, governing structures, and sizes.

Our educational goal for the collaboration was to train city representatives to conduct an urban building energy analysis for parts/segments of their building stock that they could later independently expand to the whole jurisdiction. For each city, we followed a study framework that consisted of individual pre-workshop meetings with city representatives, a joint three-day remote workshop including goal-setting and technical working sessions, and another set of individual debriefs. The workshop took place in January 2021. During the opening session, city representatives were invited to share their carbon reduction objectives for existing buildings as well as what retrofitting measures they were considering for those buildings.

**Fig. 1:**
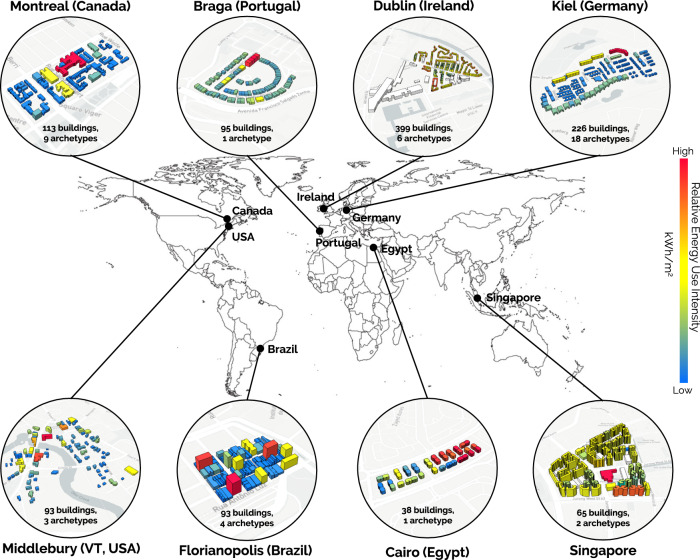
Baseline urban building energy models for the eight cities. The colour of each building indicates the relative energy use intensity compared to other buildings in the model. For example, in Singapore with two archetypes defined (viz. residential and commercial), we observe that the commercial building (in red) has a relatively higher energy use intensity than the residential buildings, which performed rather similarly, reflecting how all residential buildings in the region of interest are similar-type public housing apartment units.

We then built eight seed urban building energy models (UBEM) ranging from 38 to 399 buildings in neighborhoods for which building footprints, heights, program (usage type), and year of construction were available (Fig. 1). An UBEM is a physics-based model of buildings that estimates hourly energy use for heating, cooling, hot water, lighting, and equipment for “as is” conditions and any combination of possible retrofit upgrades. Non-geometric building properties such as construction characteristics, building age, heating, ventilation, and air-conditioning system properties were compiled for each city before the workshop (see Methods).

The concept of a “seed” UBEM was introduced for this project. A seed UBEM is a scaled-down version of a full UBEM that covers a limited part of a jurisdiction. Working with seed UBEMs (and fewer buildings) in the workshop is useful for staying nimble and supporting on-the-spot analysis. A seed model should ideally represent the city’s overall building stock—i.e., covers building typologies that represent a significant fraction of all buildings—and extend over an area that will soon undergo substantial renovation efforts. If well chosen, the seed model simulation and analysis results are indicative of the entire stock model since—with more buildings—the difference introduced stems mainly from building geometry.

The regions for the seed models were selected in consultation with participating city representatives. Florianopolis and Montreal selected typical mixed-use neighborhoods, including residential, retail, and larger commercial buildings. Braga, Dublin, Kiel, and Middlebury selected aging residential neighborhoods that are representatives of many similar neighborhoods surrounding the city core and are slated to undergo energy retrofits soon. Cairo and Singapore focused on multi-story public housing complexes that comprise most of those cities’ construction. For example, over 80% of Singaporeans live in public housing.

For each study area, we developed a baseline as well as shallow and deep retrofit scenarios based on input from the city representatives (see Results). Our research goals were to gauge the value city representatives would retain from using an UBEM-based model of their building stock and what specific building retrofit upgrades they were considering at the time from a list of possible options (see Methods). Although we provided some informal feedback at the end of the workshop to guide future development, we refrained from assuming the role of a “consultant” that provides custom-tailored, optimal solutions to each city.

In addition to building retrofits, we also predicted the maximum onsite electricity generation potential from PV assuming full rooftop utilization to provide an upper physical limit for onsite carbon emission reductions. To separate the emissions reduction contributions from building upgrades and grid decarbonization, future carbon emissions are shown as a range in the Results section, assuming current and projected future grid emissions, respectively.

This work contributes to urban-scale energy research and policy in multiple ways. Previous work on urban building energy modeling mainly focused on developing and validating simulation tools and identifying potential use cases for this new technology^11,12^. In select cases, the energy-saving potential from retrofitting existing buildings—for example, in San Francisco, CA^13^, and Venice, Italy^14^—was calculated. However, those studies do not report if and how the authors engaged with local governments. The LA100^15^ and Carbon Free Boston^16^ studies are notable exceptions where experts from a U.S. National Lab or university collaborated with the municipalities in Los Angeles and Boston to develop carbon reduction pathways using custom-built, fully integrated cross-sector models.

This paper presents the first study in which a scalable UBEM approach has been tested with multiple, diverse city representatives to understand whether local teams can learn how to use and independently apply the method and provide lasting value for participating jurisdictions. Our findings offer insight into what type of building retrofit packages energy policymakers are currently considering for their existing building stock and how resulting carbon emission reductions compare to politically motivated targets. This study also dovetails with parallel efforts to decarbonize the transportation, industrial, and electricity sectors.

## Results

### Baseline and upgrade building energy scenarios

The eight cities represent diverse cultures and climate zones where many urbanites live. In the following, we summarize the building retrofit upgrade scenarios that the city representatives formulated. The scenarios consist of combinations of building upgrades picked from a list of common technologies provided to city representatives before the workshop (see Methods). For each city, we defined two retrofit upgrade scenarios that mostly correspond to shallow (lower cost and/or easier to implement) and deep (more expensive and/or harder to implement) retrofits (Table 1). Details on how the baseline and upgrade scenarios were modeled are provided in the Methods section.

**Table 1:** Baseline model description and retrofit scenarios for the eight cities

City	Carbon emissions reduction targets	Baseline UBEM configuration	Shallow retrofit scenario	Deep retrofit scenario
Singapore	36% reduction from baseline by 2030; 50% reduction by 2050, adopted from Singapore’s emissions intensity goals^51^	65 buildings with two archetypes, primarily residential	Energy efficient lighting and appliances, as well as improved natural ventilation for commercial buildings	District cooling system, in addition to provisions in shallow retrofit
Cairo (Egypt)	No targets provided or found	38 buildings with one residential archetype	Energy-efficient lighting and appliances	Enhanced ACMV systems, in addition to provisions in shallow retrofit
Florianopolis (Brazil)	43% reduction by 2030; net-zero operational carbon by 2050 *(adopted from Brazil’s country-wide targets (originally set for 2060 during our workshop but subsequently revised by the Brazilian government for 2050 instead*^52,53^.)	93 buildings with four archetypes, mixed	Energy-efficient appliances	Enhanced ACMV systems, in addition to provisions in shallow retrofit
Braga (Portugal)	40% reduction by 2030; net-zero operational carbon by 2050 *(targets provided by city representatives)*	95 buildings with one residential archetype	Baseline model simulated with future 2080 climate and air conditioning	Improved insulation and shading devices
Kiel (Germany)	95% reduction by 2050^54^	226 buildings, 14 archetypes, mixed.	Improved insulation properties for the whole building stock	Heat pumps for space heating, in addition to provisions in shallow retrofit
Dublin (Ireland)	40% reduction by 2030^55^	399 buildings with six archetypes, mixed	Better weatherization properties	Enhanced insulation and glazing, in addition to provisions in shallow retrofit
Middlebury, VT (USA)	40% reduction by 2030 and net-zero operational carbon by 2050^56,57^	93 buildings with three archetypes, mixed	Heat pumps for space heating	Improved envelope, in addition to provisions in shallow retrofit
Montreal (Canada)	55% reduction by 2030; net-zero operational carbon by 2050^58^	113 buildings with nine archetypes, mixed	Baseline model with natural gas furnaces replacing resistance baseboard heating	Heat pumps for space heating

See Methods for quantitative descriptions of fields.

The selection process for the scenarios varied depending on the role of the city representatives, which ranged from city planners and sustainability directors to local NGOs. For some cities, a single representative “dictated” the scenarios, while other city teams were more collaborative, reflecting differences in how cities are generally governed.

Table 1 also lists carbon emissions reduction targets for buildings in each city. While some cities have separate near- and long-term goals for 2030 and 2050, others only have a 2050 goal or no goal at all. This information was provided by the city representatives and validated via official policy documents where possible. If no building-specific targets were available, we used economy-wide emission reduction targets. Braga, Florianopolis, and Montreal aimed for net-zero operational carbon emissions, meaning any remaining fossil fuel use will need to be balanced by carbon offsets or excess renewable energy generation.

Singapore, situated near the equator, has a typical tropical climate with uniformly high temperatures and relative humidity. The building stock is dominated by residential high-rises with moderately sized housing units, the ubiquitous use of air conditioning, and sound construction practices that manage to keep average household electricity use at the same level as temperate France^17,18^. The participating policymakers from Singapore’s Urban Redevelopment Authority (URA) expressed interest in equipment load reductions, the impact of pandemic-related remote working patterns on load curves^19^, as well as the energy-saving potential of district cooling systems^20^. To limit the scope of the investigation to the workshop goal of identifying carbon reduction pathways for buildings, our two upgrade scenarios for Singapore focus on the first and last areas of interest.

Cairo has a subtropical desert climate with mild winters and hot summers. The city is cooling-dominated, with increasing risks of severe heat waves due to climate change^21^. The participating UN Habitat Egypt Office presented the twin goal of keeping the population healthy without excessive reliance on air-conditioning and a focus on the widespread deployment of rooftop photovoltaics (PV). The team did not provide a carbon reduction target. Based on previous experience working in the region^22^, we tested load reductions for shallow retrofits and enhanced air-conditioning and mechanical ventilation (ACMV) equipment for deep retrofits.

Florianopolis has a warm, humid subtropical climate with warm summers and mild winters. At the time of the workshop, the city did not have any carbon reduction goals for buildings but referred to Brazil-wide targets of 43% and 100% emissions reductions by 2030 and 2050, respectively. Technologies of interest to the local team, consisting of municipal representatives and a nearby university, initially ranged from architectural interventions such as added exterior shading (fixed individual window overhangs), reduced equipment loads, and enhancements to ACMV equipment. As the workshop progressed, the team increasingly concentrated on the latter two technology upgrades as they discovered the high cost and somewhat limited impact of exterior shading in retrofit projects.

In Braga, as in other Portuguese municipalities, a sizeable number of households suffer from energy poverty^23^. Consequently, indoor temperatures routinely reach uncomfortable levels, especially during the winter^24^. Braga is further situated in a threshold climate^25^, in which air conditioning will increasingly become a necessity as summers get warmer, a challenge to households that can already barely afford energy costs. With building energy use already low, Braga’s urban planning office is most concerned about the consequences of climate change along with energy affordability and health. For the “shallow” retrofit, we therefore use a future climate file (see Methods) to quantify future energy use if most households start using air-conditioning to remain comfortable and then upgrade the building envelopes for the deep retrofit scenario. While climate change will affect energy use and emissions from buildings in all cities, threshold climates will see the most dramatic changes in building conditioning needs. These cities will experience fundamental changes in demand for cooling systems, unlike many other cities that already have widespread air conditioning today.

Kiel is one of Germany’s major maritime centers, with a sub-oceanic climate influenced by currents from the Atlantic and the North Sea. Most homes in Kiel have no active cooling, and traditionally the emphasis has been placed on adding insulation to reduce heating energy use. The Director of the Kiel Authority for Environmental Protection expressed interest in adding insulation and weatherization to reduce heating loads and energy use. These measures are employed for the shallow retrofit scenario. For the deep retrofit scenario, electric heat pumps are added to electrify the remaining heating loads, a measure in line with ongoing German efforts to reduce the country’s reliance on natural gas from Russia^26^.

Dublin has a mild climate, mainly heating-dominated, with relatively comfortable summers and cold, humid winters. Due to the age and state of many buildings, thermal envelope upgrades represent one of the most effective retrofits^27,28^. Dublin contains a significant proportion of row houses, which is why the city targets coordinated group retrofits in the form of weatherization upgrades for the shallow retrofit scenario and added wall insulation and window replacements for the deep retrofits. During the workshop, the city representative did not express interest in heat pumps as the city was assessing the feasibility of expanding the existing district heating system.

Middlebury is situated in the U.S. state of Vermont, with warm, wet summers and frigid winters. The state has a largely decarbonized electric grid with high penetrations of renewables^29^. A key concern of participating representatives from the local energy committee and Middlebury College was understanding and quantifying the potential of heat electrification and grid resilience. Technologically, these goals translate into air-source heat pump adoption for space heating (shallow retrofit) and envelope upgrades (deep retrofit) to reduce the building’s winter peak demand caused by this newly-electrified heating.

Finally, Montreal enjoys a stable, decarbonized grid from hydropower. The city mainly seeks to reduce reliance on widely used electric resistance heating due to its inefficiency. The shallow retrofit replaces electric resistance heating with natural gas furnaces, which are cheaper to operate but more carbon-intensive. Ultimately, the city hopes to convince residents to invest in ground-source heat pumps for heating (deep retrofit) powered by their clean electricity supply. Given that natural gas furnaces, once installed in place, have a lifetime of 15+ years, the former approach is not readily compatible with the city’s 2050 target.

### Energy use intensities

**Fig. 2:**
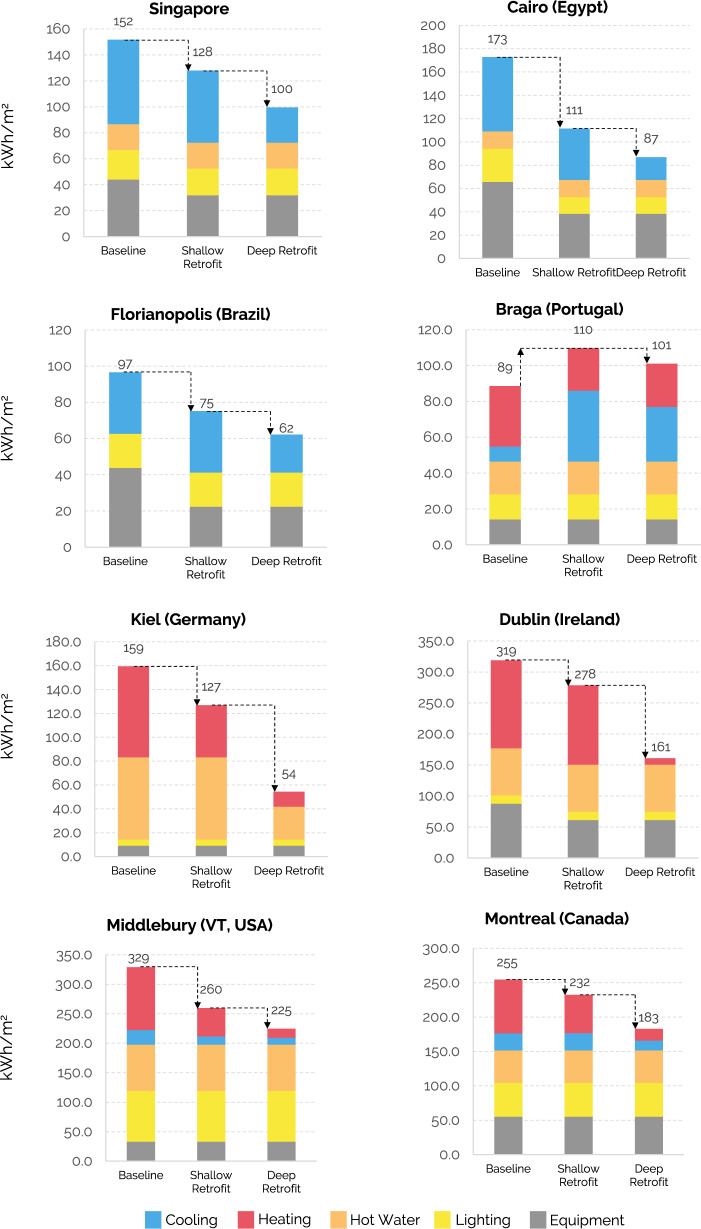
Energy Use Intensities (EUI) for the baseline and retrofit scenarios for eight cities. A baseline and two additional scenarios are defined for each city, with the colors representing energy use intensities for various end uses. Shallow retrofits naturally lead to smaller reductions in EUI, while deeper retrofits require more capital but often lead to significantly higher EUI reductions. In Braga, the “shallow retrofit” scenario accounts for a warmer climate in 2080; thus, the cooling demand greatly increases, increasing energy use.

Predicted onsite baseline energy use intensities (EUI) range from under 89 kWh/m^2^ for Braga to 329 kWh/m^2^ for Middlebury (Fig. 2). EUIs are mainly influenced by program type, climate, construction standards, mechanical systems, and urban typology. EUI subcategories for heating, cooling, lighting, domestic hot water, and equipment reflect these relationships—i.e., Cairo, Florianopolis, and Singapore are cooling-demand dominated with no heating loads. In contrast, Dublin, Kiel, Middlebury, and Montreal are heating-dominated.

In all cases except for Braga, EUIs fall for both shallow and deep retrofits. In Braga, where residents are expected to widely adopt AC units in residential construction due to a warming climate, the EUI goes up, driven by an increase in cooling energy use. The overall EUI increases by 24%, although heating demand decreases slightly due to milder winter temperatures. Retrofitting windows and fixed window overhang shading has a small impact in Florianopolis, with a simulated decrease in EUI of only 8%. Given the high cost of these measures, they were not included in the shallow or deep retrofit scenarios. In the other cities, shallow retrofits that address low-hanging fruits like reducing plug and equipment loads lead to decreases in EUI between 13% (Dublin) to 36% (Cairo). Cairo has the largest energy efficiency gains from shallow retrofits since reducing internal loads from lighting and equipment has the dual advantage of also reducing cooling loads.

Deeper retrofits naturally lead to more considerable savings, from 32% in Middlebury to 66% in Kiel. Heat pumps achieve the largest energy efficiency gains in heating-dominated climates even though these savings do not necessarily correspond to the lowest operating costs due to the widespread availability of low-cost natural gas. Kiel, for example, has significant needs for space heating and heat pumps are effective in reducing overall energy use but are historically more expensive to operate than natural gas furnaces. This is changing in 2022, with gas prices soaring due to the conflict in Ukraine, underlining the volatility of relying on fossil fuels. Dublin, like Kiel, has a high proportion of its EUI associated with heating and domestic hot water needs but opted to reduce heating loads through weatherization and insulation. However, additional steps to reduce heating emissions will need to be taken eventually. In Montreal, using natural gas instead of electricity only slightly impacts EUI but raises emissions since the hydro-powered grid is so clean. In contrast, installing heat pumps shaves off 28% from the baseline EUI, primarily from heating and cooling needs.

### Building-related peak demand

**Fig. 3:**
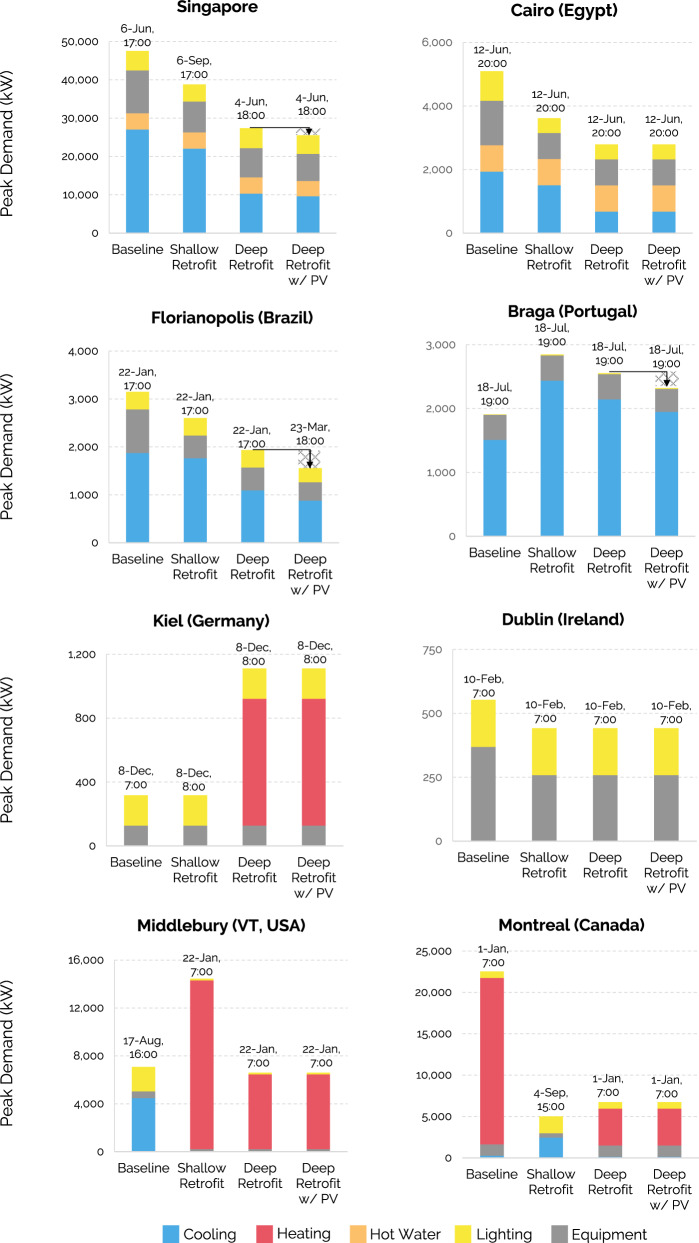
The annual building-related electric peak demand for baseline, shallow, and deep retrofit scenarios, including 100% rooftop photovoltaics deployment. The time stamp over each column marks the hour in the year when this peak occurs. We observe that the deployment of rooftop PV does not reduce the peak in many municipalities, as the peak usually occurs at the beginning or end of the day. The introduction of heat pumps for space heating significantly increases the peak in Kiel, and in Middlebury, the peak further shifts from summer afternoon to winter morning.

There is widespread consensus that decarbonizing the building sector will require the electrification of all heating systems, while the electric grid will increasingly rely on renewable energy. To realize both strategies simultaneously, it is crucial to minimize the strain that buildings place on the grid. Figure 3 accordingly shows each city’s hourly annual electricity peak demand from buildings for the three scenarios from Table 1. This value represents the hour in the year when the combined electricity demand across all existing buildings is highest. Each peak hour’s date and time stamp are included in each column. Given that most policy representatives mentioned rooftop PV to reduce onsite carbon emissions, the fourth column in Fig. 3 shows annual peaks for the deep retrofit scenario combined with PV deployment across all building rooftops. The PV simulations assume 15% module efficiency for all cities and vary by available roof area and annual solar radiation (see Methods).

In Cairo, Florianopolis, and Singapore, shallow retrofits reduce the annual peak from 9% to 29%, while deep retrofits reduce the annual peak from 39% to 55%. In Dublin, the heating is provided by natural gas in all scenarios, so the electric peak demand from buildings is driven purely by winter lighting and equipment loads and is only slightly reduced in the shallow scenario. In Cairo, Braga, and Kiel, the peaks remain around the same time of year and occur in the evening/morning for cooling/heating-dominated climates. Given the limited availability of sunlight during those times, the deployment of PV does not affect the peak loads much except in Florianopolis, where the January 23^rd^ 5pm mid-summer peak is delayed to March 23^rd^ at 6pm and reduced by 20%.

In Montreal, switching to natural gas or heat pumps for space heating would reduce the peak by a factor of three or more due to the inefficiency of electric resistance heat. In Kiel and Middlebury, introducing electric heat pumps more than doubles the peak demand from buildings, suggesting that substantial additional capacity would have to be added to the grid in these regions. In Middlebury, the buildings’ peak demand hour would further shift from the cooling-driven summer afternoon to winter mornings. However, if further combined with retrofitting measures, the peak in Middlebury could be reduced to even lower levels than the current baseline. Similarly, the widespread adoption of AC units in Braga will put a significant strain on the grid that could be somewhat prevented through deep retrofitting measures. Our findings are consistent with studies^30^ underlining the importance of buildings in grid demand management and energy policy planning. Generating units to address these peak loads—especially in the United States—typically rely on fossil fuels^31^ and can be costly to operate. Reducing peak demand from buildings thus leads to fewer fossil-fueled generation plants being brought online, decreases total annual power grid emissions, and reduces the need to build new distribution systems^32^.

Overall, our results show that the widespread use of rooftop PV will not significantly help utilities manage their building-related electricity peaks due to a temporal mismatch in production and demand. However, renewable energies will play a key role in reducing overall building-related carbon emissions as they provide a zero-carbon source of electricity to power electrified buildings.

### Carbon emissions

**Fig. 4:**
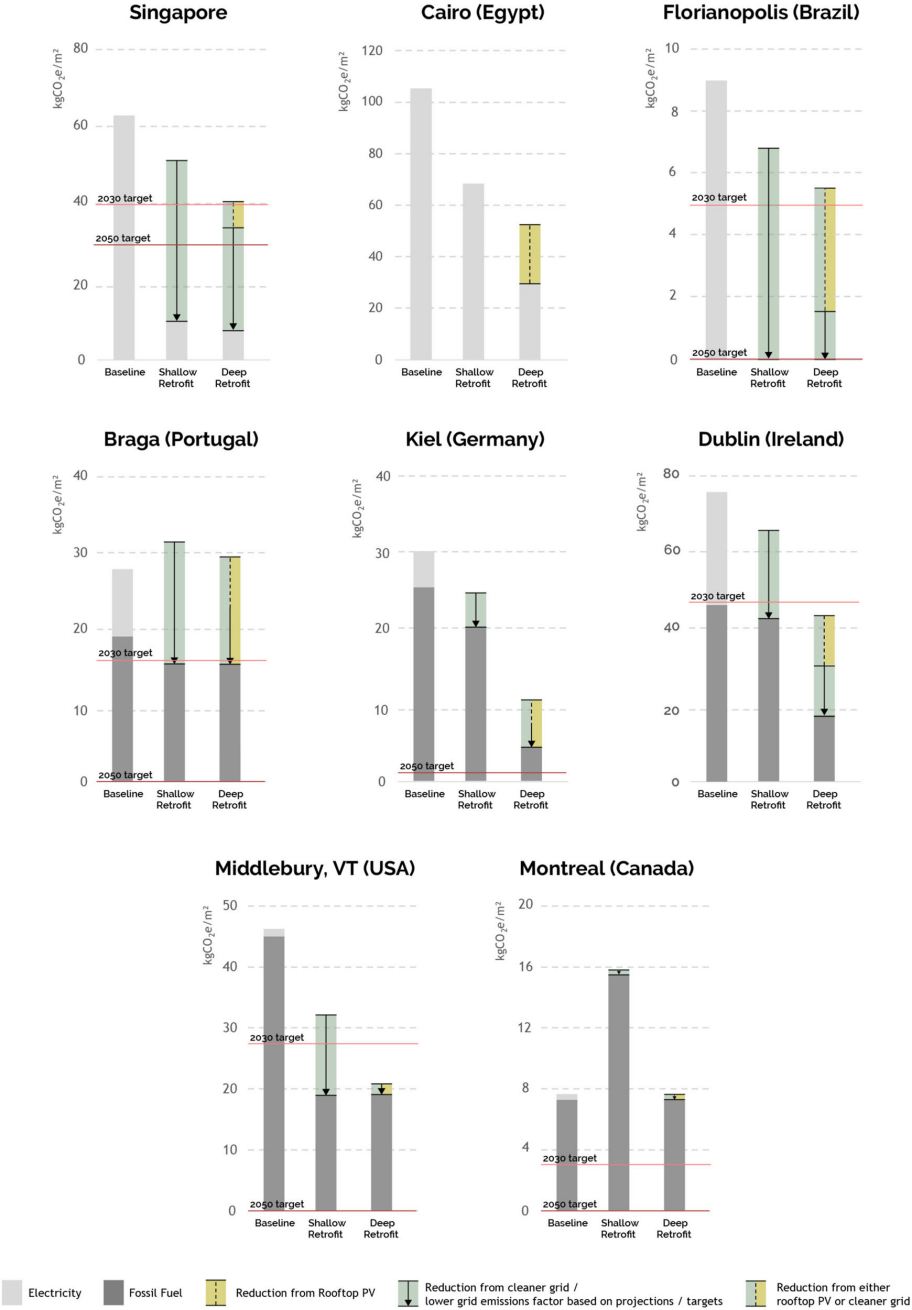
Annual carbon emissions (normalized) for baseline, shallow, and deep retrofit scenarios, with current and projected grid emissions factors and with and without 100% rooftop photovoltaics deployment. Where applicable, carbon emission targets are shown in red lines. While many cities can meet their near-term carbon emissions reduction targets, meeting their long-term targets will require grid decarbonization and end-use electrification.

Figure 4 shows annual carbon emissions for the baseline, shallow, and deep retrofit scenarios with and without PV deployment across 100% of all rooftops. For the shallow and deep scenarios, results are shown as ranges assuming 2021 and projected 2050 emission factors for electricity and fossil fuels. The underlying values were provided by city representatives, referenced from reports, and cross-checked where possible (Table 2). Note that Cairo does not have a decarbonized grid emissions target. The ranges help to separate emissions reductions from buildings and the grid. Where applicable, the city’s carbon emissions reduction targets from Table 1 are also shown. Without additional grid decarbonization efforts, total carbon emission reductions for buildings range from 13% to 36% for shallow retrofits and 34% to 84% for deep retrofits across all eight municipalities. If projected grid decarbonization plans for 2050 are fully realized, those numbers increase to 100% for shallow and deep retrofits in some municipalities.

**Table 2:** Descriptions and assumptions for the baseline archetype templates

City	Domestic hot water fuel and system	Heating fuel and system	2021 electricity emissions factor [kgCO_2_/kWh]	2050 projected electricity emissions factor [kgCO_2_/kWh]	Fuel emissions factor [kgCO_2_/kWh]	Envelope properties and schedules source	Data SOurce for Building Geometries
Singapore	Electric Resistance Boiler	N/A	0.408	0.08	N/A	Adapted from the US Department of Energy 2A Reference Buildings^59^	Building footprint polygons extracted from OpenStreetMap, heights manually measured from Google Street View and Google Earth
Cairo (Egypt)	Electric Resistance Boiler	N/A	0.61	N/A (Egypt has set 2030 interim targets but has no 2050 targets)^60^	N/A	Adapted from^22^	Building footprint polygons extracted from OpenStreetMap, heights manually measured from Google Street View and Google Earth
Florianopolis (Brazil)	Electric Resistance Boiler	N/A	0.09	0 (economy-wide including electric grid)^61^	N/A	Provided by participating academic partners from Universidade Federal de Santa Catarina	GIS Shapefiles with attributes provided by participating academic partners from Universidade Federal de Santa Catarina
Braga (Portugal)	Natural Gas Boiler	Fuel-Fired Furnace	0.237	0^62^	0.369	Adapted from^45^	GIS Shapefiles with attributes provided by participating academic partners from the Instituto Superior Técnico
Kiel (Germany)	Natural Gas Boiler	Natural-Gas Furnace	0.275	0^62^	0.18	Based on TABULA^44^ Germany data provided by academic partners from Christian-Albrecht University of Kiel	GIS Shapefiles from municipality provided by participating academic partners from Christian-Albrecht University of Kiel
Dublin (Ireland)	Electric Resistance Boiler	Fuel-Fired Furnace	0.325	0^62^	0.205	Adapted from^27^	GIS Shapefiles with attributes provided by participating academic partners from the University College Dublin
Middlebury, VT (USA)	Natural Gas Boiler	Fuel-Fired Furnace	0.011	0 (by 2035)^63^	0.247	Adapted from the U.S. Department of Energy 6A Reference Buildings^59^	GIS Shapefiles from the Vermont Open Geodata portal with attributes provided by the town planning office
Montreal (Canada)	Natural Gas Boiler	Electric Baseboard	0.0025	0 (by 2035)^64^	0.15	Adapted from the US Department of Energy 6A Reference Buildings^59^	GIS Shapefiles with attributes provided by participating academic partners from Polytechnique Montréal

Emissions factors are provided by the city representatives while the projected emissions factors are based on currently published political goals, which may or may not reflect physical reality. The values are based on national or EU-level targets to reflect the interconnectedness of the grid. Some jurisdictions have earlier zero emissions targets (e.g., the U.S.) but 2050 dates are shown for all. Heating fuel varied by city and therefore the emissions factor for the fuel varies. Finally, Singapore does not have projections for a medium or longer-term grid emissions factor (GEF), as verified with staff from the Energy Market Authority (EMA) of Singapore. The projected GEF is derived by linearly interpolating historical average operating margin (OM) emissions factor from 2005 to 2018 for 2050.

Singapore’s building energy use is modeled as all-electric, and the projected grid emission reductions would help it surpass its 2050 target for both shallow and deep retrofits. Much of the grid decarbonization will need to come from off-site sources as rooftop solar can only contribute to emissions reductions for a small part of the investigated residential high-rise buildings that make up much of Singapore’s building stock.

Cairo has no publicly available grid emissions reduction plans (nor emissions goals), and the rooftop solar production potential can reduce current emissions by 21% from baseline.

Florianopolis has the potential to meet its 2050 target through Brazil’s overall goal of a fully decarbonized grid. Over 80% of those reductions from the baseline can be realized onsite through deep retrofits and rooftop PV.

Braga’s rooftop solar potential is substantial and can contribute all the carbon-free electricity needed to meet its electrical needs in the deep retrofit scenario. However, achieving their 2050 targets will require the electrification of all other end uses. The situation is the same in Kiel, Middlebury, and Montreal, where the grid is already largely decarbonized and/or rooftop PV could cover the remaining onsite electricity demand. While traditional net-zero analyses assume that onsite fossil fuel consumption can be offset by rooftop solar, this accounting practice does not work when the local grid is fully decarbonized. Given that domestic hot water contributes substantially to these cities’ energy use (Fig. 2), it is curious that none of them opted for domestic hot water heat pumps. It is crucial that municipalities embrace a fully decarbonized system, whether fully electrified or through green hydrogen or some other carbon-neutral fuel.

In Dublin, the combination of rooftop PV and the projected decarbonized grid can more than halve the remaining emissions in the deep retrofit scenario. Given this decarbonized grid, electrification of the heating system could help Dublin meet a future net-zero target.

### Post-workshop follow-up

During the workshop’s final day, all teams suggested that the UBEM approach could support their jurisdiction’s efforts to reduce building-related carbon emissions. In September 2022—twenty months after the workshop—we contacted all eight city representatives again to understand “what (if any) activity, follow-up modeling efforts, use of the results, discussion, legislation/policymaking, or any other outcome [had] resulted from the workshop.” Seven out of eight representatives responded to our request and reported the following activities.

In Braga, to facilitate further use of UBEMs, the local participating partner (Instituto Superior Técnico of Lisbon) developed a building template library for all of Portugal, tested in another workshop in 2022 involving representatives from three additional Portuguese cities: Porto, Coimbra, and Lisbon.

The Climate Action Coordinator for Dublin shared that the city secured funding from the national government—through the Public Sector Innovation Fund—to “further utilize UBEMs to model retrofit options.”

In Florianopolis, the study became part of a guiding principles report for public policies. This report was developed through the Efficient Cities Project of the Brazilian Council for Sustainable Construction (CBCS) to advise the Municipality of Florianopolis in setting up its energy efficiency program.

The University of Kiel, in collaboration with Shell Germany, built an UBEM of the whole city that contains around 36,000 buildings. The city is interested in using the data to inform the management of its district heating network and future incentive programs.

Following the workshop, a team from Middlebury College expanded the seed UBEM to the entire town of Middlebury. The model has grown further and now covers all of Addison County’s 23 towns, with approximately 12,000 buildings. A representative survey to better characterize the building stock is ongoing to adapt the U.S. Department of Energy’s building templates to rural Vermont.

The Université de Montréal, in collaboration with the municipal government, has developed a “virtual island” model of the whole metropolitan area. The project is funded by the Institut Trottier de l’Énergie, and efforts are underway to calibrate the model using select measured data and develop plans for a heat-sharing network in neighborhoods undergoing major redevelopment.

In Singapore, students from the National University of Singapore have expanded the seed model, focusing on solar energy potential for high-rise buildings.

## Discussion

### Policy implications

While many cities recognize the urgency to reduce carbon emissions of their existing building stock and have established ambitious targets, municipal representatives struggle to define clear technology pathways that they can communicate to their constituents. For cities aspiring to reach net-zero carbon emissions—such as Braga, Florianopolis, Kiel, and Montreal—a full-scale implementation of what their representatives consider a deep retrofit along with deployment of PV on all rooftops can only reach this goal for existing buildings if their grid is decarbonized at the same time and heating and hot water end uses are fully electrified^33–35^. This need extends to cold climates where heat pump manufacturers now also offer viable solutions^36^. However, in cities like Montreal, owners thinking of adopting heat pumps now face two barriers: it is currently cheaper to heat a building with natural gas, and there is still widespread (if disproven) skepticism as to whether the latest generation of air-source heat pumps can reliably heat a building in such a cold climate^37^. As a transitional solution, existing electric resistance heating systems in Montreal could remain in place and back up newly installed heat pumps if needed. Otherwise, owners who decide today to switch to natural gas-based heating will likely remain with that technology for decades^38^. Most participating municipalities in our study also disregarded the remaining fuel use from domestic hot water, which faces the same dilemma as the electrification of space heating but (at least for our study participants) currently seems to receive less attention. A reason for this may be that domestic hot water heat pump installations in cold climates remain somewhat rare.

As mentioned in the introduction, technology adoption over time is a key missing factor in this study. Estimates for building retrofitting rates, which include non-energy related modifications such as updating a bathroom or adding an extension, tend to hover at around 1% per year^6^. This means that cities that can theoretically meet their 2030 targets through the tested technology pathways would need to instantly boost the annual retrofit rate for their deep retrofit scenario to 12.5% to reach this goal. This suggests that municipalities currently work at implementation rates that are an order of magnitude too low.

Figure 4 highlights the tight relationship between buildings and the electric grid, showing they must be decarbonized together. While municipalities are probably in a better position to help their constituents to renovate their buildings, only utilities understand the impact of such changes on the grid along with other trends, such as the widespread adoption of electric vehicles. It therefore seems that rather than working with a single cross-sector carbon reduction target, cities need specific guidance on how much savings their building stock needs to accomplish and at what time. Our workshop finding shows that UBEM-based approaches can help implement those building-specific targets.

### Workshop lessons

Our three-day workshop experiment and post-workshop survey confirm the widely reported political momentum among city governments to reduce carbon emissions. Municipal representatives implementing carbon emissions reduction targets generally appreciate data-driven methods to guide their policy development. Participating cities without previously established carbon emissions reduction targets particularly benefited from the workshop as the UBEM simulations can help them to link targets to a particular set of measures. Florianopolis, for example, reported its intention to use the results from the workshop to establish dedicated carbon reduction goals for existing buildings.

Another workshop finding is that each city’s specific technology measures significantly vary due to climate, political, and economic boundary conditions, and the state of existing buildings. There is no one-size-fits-all approach for the built environment. It should be stressed that while the technology measures modeled and explored in this study consider local building stock characteristics, they would not necessarily deliver the most cost-effective decarbonization or EUI reductions nor move the city/municipality most expediently towards its stated carbon goal. This is not surprising. Decades ago, individual building energy models were developed to help design teams identify the most suitable combination of energy conservation measures for a particular building project.

While the seed UBEMs used in this workshop provide a first benchmark result, we caution that cities with non-homogenous building stocks will need to model their entire building stock. The required effort mainly consists of additional simulation time and data storage, i.e., cost, rather than human resources or additional expertise. Fortunately, four out of eight participating municipalities of varying size (Dublin, Kiel, Middlebury, and Montreal) did manage to secure public or private funding to make UBEMs an integral part of managing local energy infrastructure, such as district heating systems or establishing retrofit incentive programs. We therefore conclude that this approach is scalable and encourage municipalities worldwide to conduct similar data-driven studies to establish baselines and predict the saving potential for various technologies. The resulting policy plans, which describe what upgrades need to happen in which type of buildings, can be effective for political consensus building as individual homeowners, who ultimately have to pay for implementing those changes, can understand how their contributions fit within a larger context. Such an analysis also ensures that cities do not overlook energy use from, for example, domestic hot water.

Given the plethora of tasks that municipal government workers face today, it seems likely that more NGOs or sustainability consulting offices will start offering UBEM-based carbon reduction strategies to city governments to inform long-term energy policy.

Thinking beyond these introductory workshops, which provide a technological pathway to align policy targets with concrete actions, city governments will need to engage in a series of follow-up exercises. These include carefully considering the costs to homeowners of the desired upgrades, raising awareness of existing incentive programs among eligible citizens, and potentially lobbying for new subsidy programs that ensure retrofit measures are implemented across the demographic spectrum. Training a local workforce to implement those changes at fair costs is also vital. Finally, efforts to address emissions from new construction will also be critical to achieving emissions targets.

Politically driven carbon reduction goals for existing buildings are currently somewhat disconnected from technical realities in terms of both the extent of considered upgrades and the speed of implementation. We demonstrate that recently developed urban building energy modeling workflows have matured to a point at which they can be widely applied with low effort and offer actionable information for municipal decision-makers. With these tools, municipalities can develop financial incentive programs that persuade owners to upgrade their buildings while training a workforce that can implement these measures. They also ensure that no emissions-reducing interventions are left on the table in the quest to achieve ambitious but necessary emissions-reduction goals.

## Methods

We deploy a consistent study framework across eight cities. Specifically, we first partner with representatives from the eight cities to identify policy objectives and carbon emissions reduction goals. We then work with each city to identify prototypical regions representing the local building stock (seed neighborhoods). Next, we gather geometric data such as geographical information system (GIS) files containing building footprints and building heights, as well as non-geometrical properties of the building stock, including but not limited to construction properties, window-to-wall ratios, mechanical system types, and occupancy profiles. We retrieve weather data for each city from public repositories^39,40^.

These inputs are combined to construct seed urban building energy models for each city and run a baseline operational energy simulation of existing conditions. We then implement shallow and deep retrofit scenarios in two derivative seed UBEMs and run multiple simulations to obtain energy use, peak demand, and carbon emissions. Finally, we present the results to the city representatives for feedback and discussions. Further details on select steps are provided below.

### Carbon reduction goals

Although the participating cities differ in size, climate, demographics, urban typologies, and building characteristics, most cities in our study have at least economy-wide carbon emissions reduction strategies or climate action plans. These targets are broadly in line with the Paris Climate Agreement, with most plans having timelines including a near-term target and a longer-term goal aiming for economy-wide net-zero emissions by 2050. However, only five out of the eight participating cities indicate that they have a detailed carbon inventory, and only four have carbon reduction plans specifically for buildings. Most participating cities originally derived their buildings’ carbon reduction goals and targets using a mix of in-house teams, government agencies, and external consultants. Only two cities reported having previously used data-driven methods to inform their targets.

### Climate data

An annual weather file is needed for every city to characterize local climate conditions. We use typical meteorological year (TMY) weather files that are freely available online for each of the eight jurisdictions. TMYs are text files that include location-specific attributes such as longitude, latitude, elevation, monthly average ground temperature, historic hourly dry bulb temperature, relative humidity, solar radiation, and wind speed^41^. To study the impact of climate change on the city of Braga, we use the CCWeatherGen tool^42^ to generate a morphed weather file for 2080 that represents the potential future climate in the region.

### Building archetypes

To model an existing building stock, the buildings must be divided into similar groups or archetypes. For all archetypes, so-called building simulation templates must be developed. These templates contain non-geometric building information that ranges from construction practices to usage schedules, setpoints, and HVAC system performance. This is the most challenging part of the analysis, as it requires expert knowledge of each city’s current and historic construction practices. To tackle this task, it is customary to break the building stock into archetypes by the construction period and usage type (i.e., residential, commercial, retail, etc.). For the U.S. and Canada, the U.S. Department of Energy offers detailed building descriptions for 16 program types and 16 climate zones in the form of Commercial Reference Buildings^43^ which were used for Middlebury and Montreal. For Dublin, Buckley et al. (2021)^27^ recently proposed and validated a workflow to convert stock data from the European Tabula Project^44^ into archetype templates used for this analysis^28^. A similar approach was adopted for Kiel, as Germany had also participated in the Tabula project. For Braga, we used UBEM templates previously developed for the Portuguese building stock by Monteiro et al.^45^. For Cairo, we used validated building templates from a previous project in Kuwait^22^. In Singapore, we started with the U.S. Department of Energy Reference Buildings for Climate Zone 2A^43^ and worked with local building science experts who were part of the city’s modeling team to adjust them to the local context. In Florianopolis, we relied on templates provided by building simulation experts from the Federal University of Santa Catarina, who also participated in the workshop. Table 2 summarizes key information for all eight cities’ baseline archetypes and GIS data. Table 2 also documents the 2021 and projected 2050 electricity emissions factors used to calculate all carbon emissions from electricity consumption in each jurisdiction.

### UBEM development

Urban building energy modeling (UBEM) is a physics-based approach to simulate the thermal performance, space conditioning loads, and energy use of multiple buildings on the urban scale^11^. The authors previously developed a web-based tool (UBEM.io)^46^ for multiple use cases, that rapidly generates urban building energy models based on GIS shapefiles containing building footprints, building heights, and program types. UBEM.io comprises a front-end built using JavaScript (and the React JavaScript library) for user interaction and inputs. A backend developed with the Python programming language provides the application programming interfaces (APIs) and functionalities. It also synchronizes with a building template library, allowing users to assign building parameter templates to characterize the buildings’ physical properties, mechanical systems, occupancy profiles, and other attributes.

The baseline urban building energy models for all eight cities are constructed via UBEM.io using the input GIS, TMY, and template files described above. We subsequently conducted the urban building energy modeling and building performance simulations using the Urban Modeling Interface (UMI)^47^, a simulation plugin for the Rhinoceros3D computer-aided design (CAD) environment. UMI utilizes the EnergyPlus simulation engine^48^ to simulate space conditioning (e.g., heating, cooling, ventilation), equipment, and other loads and their associated energy use. EnergyPlus is a whole-building console-based energy modeling engine that implements detailed physics-based calculations for heat transfer, air, and other thermal metrics.

While no measured building energy data was available for the seed UBEMs, according to a comparison of multiple UBEM studies^49^, our uncalibrated baseline UBEMs with carefully selected templates provide sufficiently accurate information to predict annual energy use and (more importantly) estimate energy and carbon emission savings from various retrofit measures at the neighborhood level. This is because simulation errors for individual buildings tend to cancel out at the urban scale.

### Retrofit scenarios

After generating the baseline seed UBEMs, we build shallow and deep retrofit archetype templates based on the stakeholder input documented in Table 1. Before the workshop, city representatives were given a list of common building technologies in Table 3. Tables 4 and 5 provide key simulation assumptions for the shallow and deep retrofit upgrades. Resulting baseline and upgrade scenarios are then run with results reported at hourly intervals. The cumulative yearly energy consumption is used along with the various emissions factors to report yearly carbon dioxide emissions, while the hourly results are used to find the peak demand. This is the hour in the year when the electricity consumption is maximal across all electricity end-uses in the buildings, setting the yearly peak demand value (in kW).

**Table 3:** Pre-workshop survey questions provided to municipal representatives

Which building retrofit technologies is your city/municipality most interested in?
Control strategies (e.g., smart thermostats)
District energy systems (e.g., low-temperature district heating)
Envelope retrofitting (e.g., façade, roof, windows, weatherization, etc.)
Lighting (e.g., LEDs)
Heat pumps (e.g., geothermal and/or air -source, etc.)
Passive design strategies
Solar photovoltaics
Other

**Table 4:** Lighting power densities and equipment loads for the baseline, shallow and deep retrofit building simulation templates

		Lighting power density [W/m^2^]			Equipment power density [W/m^2^]		
City	Archetype	Baseline	Shallow retrofit	Deep retrofit	Baseline	Shallow retrofit	Deep retrofit
Braga	Commercial	NA	NA	NA	NA	NA	NA
	Residential	5	5	1.5	13	13	6
Cairo	Commercial	8	4	4	12	7	7
	Residential	8	4	4	12	7	7
Dublin	Commercial	NA	NA	NA	NA	NA	NA
	Residential	5	5	5	10	7	7
Florianopolis	Commercial	9.13	9.13	9.13	24.2	11.3	11.3
	Residential	5	5	5	6	4.5	4.5
Kiel	Commercial	1.5	1.5	1.5	2	2	2
	Residential	1.5	1.5	1.5	2	2	2
Middlebury	Commercial	12	12	12	8	8	8
	Residential	2.6	2.6	2.6	10	10	10
Montreal	Commercial	12	12	12	8	8	8
	Residential	7	7	7	4	4	4
Singapore	Commercial	11.5	8	8	14.5	10.5	10.5
	Residential	9	5	5	14	8	8

Values shown are the mean across all that types for cities with more than two templates.

**Table 5:** Average heating and cooling COPs for the baseline, shallow, and deep retrofit templates

	Heating COP			Cooling COP		
City	Baseline	Shallow retrofit	Deep retrofit	Baseline	Shallow retrofit	Deep retrofit
Braga	0.9	0.9	0.9	NA	3	3
Cairo	NA	NA	NA	2	2	4.5
Dublin	0.65	0.9	0.9	NA	NA	NA
Florianopolis	NA	NA	3	3	3	5
Kiel	1	1	3.5	NA	NA	NA
Middlebury	0.9	2	3	2	3.5	4
Montreal	1	0.95	4	3	3	4
Singapore	NA	NA	NA	3.23	3.23	6.5

### Photovoltaic modeling and simulation

**Fig. 5:**
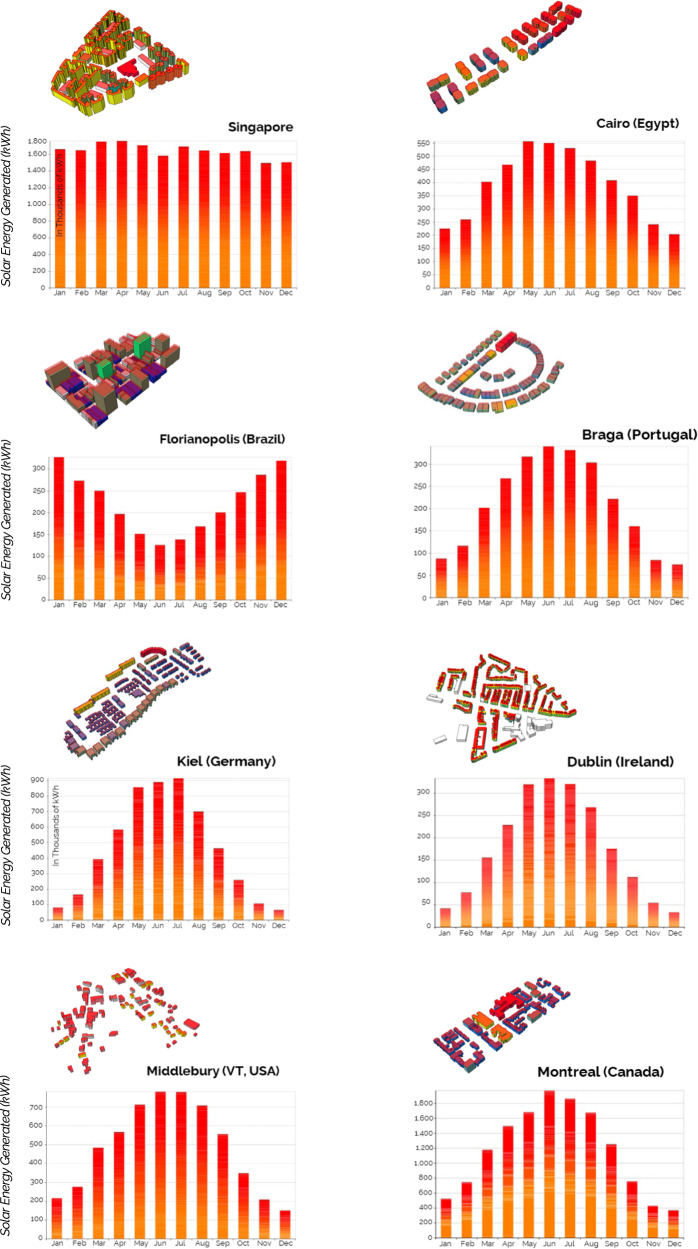
Solar energy analysis for the eight cities and annual solar energy yield. To analyze solar energy potential, we designed a custom visual programming script in the Rhinoceros3D CAD and Grasshopper visual programming/scripting environment. Specifically, our script extrudes rooftop areas as horizontal surfaces with surface normal pointing in the direction of the z-axis. Based on the areas, the weather file, potential shading, and efficiencies of the solar panels for each city, we run the modeling and simulation to derive the annual energy yield for each region (in kWh). The bar graphs show the total solar energy generated over a year for all rooftops in the seed neighborhoods.

To simulate rooftop PV potential, we rely on the EnergyPlus PV module, invoked via ClimateStudio^50^, an environmental performance and analysis tool for the Rhinoceros3D CAD environment. The simulations assume PV module efficiencies of 15%, with modules installed on all rooftop areas in the seed UBEMs. The calculations consider shading from neighboring buildings when estimating potential electricity generation from PV. Figure 5 shows the resulting monthly solar energy yield for each municipality.

## Data Availability

Certain building-specific data provided by the city representatives for this study are confidential due to privacy concerns, but otherwise, data for the seed models are available upon request.

## Code availability

UMI^47^ and UBEM.io^46^ are publicly available and free to use with select code repositories and custom scripts available upon request. The method can be replicated and is scalable for any other city or municipality.
